# The Relation of Veterinarians to the Public Health as Meat- and Milk-Inspectors1Read at the March meeting of the Veterinary Medical Association of New York County.

**Published:** 1898-05

**Authors:** H. D. Gill

**Affiliations:** New York College of Veterinary Surgeons, New York


### THE JOURNAL OF COMPARATIVE MEDICINE AND VETERINARY ARCHIVES.

VOL. XIX. *MAY, 1898.* No. 5.

### THE RELATION OF VETERINARIANS TO THE PUBLIC HEALTH AS MEAT- AND MILK-INSPECTORS.[1]

By Dr. H. D. Gill,
NEW YORK COLLEGE OF VETERINARY SURGEONS,
NEW YORK.

DISEASE frequently results from the consumption of unwholesome meat and milk. It becomes, therefore, a natural presumption that sanitary authorities should take such means as are necessary to prevent the sale of such food.

In this paper I wish to show the important relation of veterinarians to the sanitary authorities as guardians of the public health, and also to point out some of the necessary safeguards that should be placed upon the sale of animal foods.

I shall speak of milk first, as the greatest danger of the transmission of disease is from this source. Milk forms the principal diet of infants and invalids, and is generally consumed in its raw state, while meat is more or less cooked. By the inspection of milk one cannot determine the condition of the animal from which it was derived, therefore control and the inspection of cows, together with preventive restrictions against the contamination of their products, become absolutely necessary. This is also true of meat-inspection, for there are some conditions that affect the quality of the meat that cannot be detected after the animal is slaughtered.

*Milk.* Milk is unfit for use as food under several conditions :

*a.* When derived from animals in condition unfit to furnish milk, as certain physiological conditions, like advanced pregnancy or calving period.

*b.* When it contains some infectious matter.

[1] Read at the March meeting of the Veterinary Medical Association of New York County.

c. When adulterated. Adulteration may consist either in the addition of some substance to the milk, as water or coloring matter, the subtraction of some constituent like cream, or the addition of preservatives, such as borax, etc.

The determination of adulteration obviously lies within the domain of chemistry.

Let us turn to the various possible ways in which infection may be introduced. These ways may be summarized as follows:

1. From the animal supplying the milk.
2. From the persons who handle the milk.
3. From various substances that come in contact with or are introduced into the milk.

In how many ways the animal may be the source of infection it would require too long a time to describe; but tuberculosis and other constitutional diseases, local disease (external and internal) of the udder or teat, sufficiently suggest abundant causes of infection. These are causes which can be determined, we must admit, only by the veterinarian.

Of no less importance is the inspection and direction of the care which should be given the animals as regards their food and water and as regards the ventilation and sanitary arrangements of the stables.

Of the sources of infection from the person, such as association with an infectious disease, like diphtheria and scarlet fever, I will not speak more; but I would call to your attention the necessity of care in the entire milking process, the abundant opportunity for infection occurring then, and point out that so far as the process is one involving the handling of animals and their proper treatment, it is one which should also be directed by competent, that is, by veterinary advice.

Aside from the animal supplying, and the person handling the milk, great danger of infection arises from exposure, particularly while milking. Dust from hay, etc., manure, and dirt thrown by the tail and hoofs of cows while fighting flies and mosquitoes, and many other conditions of the dairy go to make up the principal sources of milk contamination. This makes the maintenance of a separate milking-room indispensable.

*Meat.* The conditions requiring the exclusion of meat from market are numerous. Some of the most important are tuberculosis, meat infested with trichinæ and other parasites, hydatids, hog-cholera, etc. Too often inspections have been made by persons who have not been trained to observe and exclude more than the most obvious diseased conditions, and whose only ground for fitness seems to have been that they had been trained as butchers.

That such inspection belongs to veterinary medicine I think no one will dispute, and it is of such magnitude and importance as to make necessary for its proper performance in large cities the establishment of a separate division in the Health Department, to be under the supervision of a competent and experienced veterinarian.

The United States Department of Agriculture some years ago established a Bureau of Animal Industry, virtually a veterinary bureau; the chiefs and inspectors are required by law to be graduated veterinarians. Their most important duty is the inspection of all meat to be used for export. The law under which they act directs that meat for interstate trade shall also be inspected, but for want of a sufficiently large appropriation this is only done in some of the largest abattoirs.

The carcasses, when healthy, are stamped or tagged, and those unfit for food are condemned. To-day nearly all of our Western beef bears the mark of a government inspector, and naturally demands the highest price.

*Local Inspection.* At our large city abattoirs meat-inspection is in force, both governmental and local; but it is from the suburban or country slaughter-houses we must look for the most danger. At such places there is no inspection, and the result is that a surprisingly large amount of diseased meat is shipped to our city, and purchased and consumed by the poorer class of people, who are compelled to buy cheap meat or go without. In this manner sickness and the death-rate are materially increased.

To give you an idea of some of the common practices I will cite the following: A farmer has one or a number of sick cows that he fears may die; he therefore slaughters them and ships the meat to a New York commission merchant, who sells it for his account, or he may sell or trade the cattle to a drover, who follows the same course.

Under the present conditions New York offers an excellent market for the sale of such meat. No matter how perfect or rigid an inspection is carried on in our city, we can never guarantee the inhabitants pure and wholesome meat until the sanitary authorities exclude uninspected meat.

To better appreciate the relation and the duties of the veterinarian to the public health, I have prepared the following summary:

*Milk Inspection.* This should include:

1. The testing with tuberculin of all cows supplying milk to the city. This includes tagging for identification.

2. The inspection of cows for other diseases or conditions that might render the milk unfit for use.

3. The examination of water used both for drinking and for cleansing utensils.

4. The inspection of food.

5. The inspection of stables. This includes arrangement of stalls, air-space, ventilation, drainage, cleanliness, etc.

6. The care of animals. This includes the grooming, exercising, care of hoofs, etc.

7. The care of utensils used in milking. These should either be washed in boiling water containing soda or potash, or, better still, sterilized.

8. Inspection of dairy employés; particular attention should be paid to the cleanliness of the hands and clothes of attendants, and not under any circumstance should they be employed if they have been in contact with, or themselves have, any infectious or contagious disease.

9. The care and handling of milk. Milk should not be exposed, and while cooling should be covered with a layer of cotton between two pieces of wire gauze; this will allow evaporation, and prevent infection. All milk-bottles should be sterilized, and should be filled and sealed under antiseptic precautions.

*Inspection of animals within the city limits.* This includes:

*a.* The physical examination of all animals for contagious and infectious diseases, many of which may be transmitted to human beings. The most dangerous of these are the following:

In cattle: Tuberculosis.
In horses and mules: Glanders and farcy.
In swine: Cholera and trichinosis.
In sheep: Anthrax.
In dogs: Hydrophobia.

*b.* The testing of cattle with tuberculin for the detection of tuberculosis. This includes the tagging for identification.

*c.* The testing of horses and mules with mallein for the detection of glanders and farcy.

*d.* The examination of meat-producing animals at slaughter-houses before they are killed. This is important, because there are some conditions, as fever, fatigue, exhaustion, starvation, and excitement, affecting the quality of the meat, that cannot be detected after the animal has been slaughtered.

*e.* The inspection of animals entering the city stock-yards, freight and express depots, boat-landings, and slaughter-houses.

*f.* The transportation of animals. This includes driving or carting animals through the city, disinfection of trucks, etc.

*Inspection of Meat.* This includes the inspection of the meat of animals killed :

*a.* Outside of the city limits.

*b.* At slaughter-houses.

*c.* In butcher shops.

All animals and carcasses inspected should be stamped or tagged. Meat unfit for food should be destroyed.

No meat should be allowed to enter the city unless it bears the stamp or tag of a government inspector, or a certificate from a reputable veterinarian stating, under oath, that he inspected the animals and that he found no evidence of disease before or after death.

The shipper must notify the Health Department, stating when and how shipped, so that the meat can be again inspected and tagged or stamped at the terminal, express, or freight office before being put on the market.

It would then be unlawful for any butcher to sell meat that does not bear the stamp of a meat-inspector.

*Supervision of slaughtering and handling the dressed meat.*

*a.* To see that this is carried out in a cleanly manner and that the meat is not contaminated by carelessness and filthy surroundings.

*Veterinary supervision of animals owned by the health department.*

*a.* Working horses (inspectors, ambulances, disinfection, etc).

*b.* Examination, care, and autopsy of calves and heifers used in the production of vaccine virus.

*c.* Veterinary examination, care, and autopsy of animals used for the production of antitoxic serums. This includes examination and purchase of horses, injecting toxins, surgical and medical treatment after injecting, and the bleeding of the same.

*Inspection and autopsy of dead animals.*

*Disposition of carcasses of animals dying from contagious diseases.*

*a.* Inspection of dead animals at dock to determine prevalence and location of disease.

*b.* Inspection of dead animals in street.

*c.* Inspection of hides.

*Disinfection of stables.*

### DISCUSSION.

Dr. Austin Peters: It gives me pleasure this evening to listen to Dr. Gill's paper and to note what he says about meat- and milk-inspection. I think his ideas are all right, but at the same time I think that there would be a great deal of difficulty in carrying them all out.

Five years ago last fall the New York State Board of Health became very much interested in the inspection of cows' milk and the possible danger to human beings from tuberculosis. In 1892 they had a bill passed appropriating $5000 to inspect dairy cattle in New York State. When infected cattle were destroyed the owner could claim the cost from the State. I was appointed chief inspector, and with others inspected the animals of Westchester and Orange Counties, about 10,000 in each county, and nearly all in Long Island City; we gave each cow a physical examination, and the result was that the claims to be paid amounted to $20,000. This scared them so that they appointed a commission, and they have not done very much since in that line.

In Massachusetts, by law, an inspector of animals must be appointed in every city and town in the State, to inspect the animals in the slaughter-houses, also to go around among the stables and examine the cattle. There is no State Veterinarian, and the work of the dairy inspection is in the hands of the State Board of Cattle Commissioners. They inspect once a year, and the result has been that the last two years the Cattle Commission had $300,000 to spend, and last year $250,000, and this year there is a very strong feeling of economy in the State, and they are going to try to have only $150,000. We killed about 5400 head of cattle last year under the tuberculin-test; we had about 10,000 returned as suspicious. Of these there were about 4000 found to be healthy, and about 5400 were quarantined. This saved $185,000. If an animal is quarantined by an inspector under suspicion, an agent of the Board is sent to examine the animal. As a rule, the agent tests with tuberculin, and if condemned the owner is allowed an amount not to exceed $60.

The law says that any animal that is diseased is unfit for human food, and it says that tuberculosis is a disease that is dangerous to the human family, but it seems to me tuberculin is an extravagant way to work on. There are a lot infected in the mediastinal gland which are killed and used for fertilizer.

The law is now changed, so that we do not pay over $30; but it is uncertain whether this limit as to value will continue, as there are a great many cases where people are keeping cows to put them in quarantine so as to sell them to the State and get the price allowed. I think that where a cow is condemned there should be a system whereby the carcass could be used as a fertilizer and the money received for it be returned to the owner.

I think that in all our States the rules for meat-inspection should be made to conform with the United States Bureau of Animal Industry. They have meat-inspection under certain rules throughout the country. If the animal has a slight localized lesion it is passed; if it is generalized the carcass is thrown away.

I think that as far as public health goes we have to look at it from two standpoints: First, the possible danger from cows that are tuberculous; the other, that it is a contagious disease. I think that if we killed badly diseased animals on physical examination, those which have tuberculous udders, for instance, it would be cheaper. A cow may have actinomycosis or inflammation of the udder, and she will produce germs which are parasitic in character, which, of course, are injurious to the milk. It is very necessary that the conditions under which milk is produced should be known. For that reason I think that a fixed method of dairy inspection is very important.

For the past year Dr. Theobald Smith has been doing a good deal of work in this line. He says there is a difference between bovine tuberculosis and human. It is found that the bacillus from human is more slender than that of cattle, which is thick and harder to cultivate. When human bacillus is injected between the ribs it produces a slight localized lesion or small abscess, whereas the bovine germ injected in the same way has produced in two weeks or a few months generalized tuberculosis. Some counteract quicker than others.

As a great deal of milk comes from outside of the State it is doubtful what the result of inspection would be; the only way would be to license each dairyman, who would have to have a certain standard to go by. In dealing with contagious diseases and using the tuberculin-test, we have t have the co-operation of the owner, for if he is careless about disinfection we cannot depend on him very much.

Dr. Pearson: This subject was under discussion at the U. S. V. M. A. meeting at Des Moines three years ago, and at that time there were some who advocated it; but one of the speakers doubted the efficiency of these sanitary measures and of the power of veterinarians to control them.

In approaching this subject we must not do it as the man who was in danger, and compelled to pray, and as he doubted the existence of a God, he said: "Oh! God, if there be one, in heaven, if there is one, protect me, if you have power." We do not want to go at it in that way. We want to know, if there are dangers, how they can be avoided. Of course, there are some who will say that veterinarians recommend these measures because they will be employed, because they will receive money, and, of course, it may be that motives of that kind will be found; but there are other reasons, and because they are the best experts on these questions.

Meat-inspection is nothing more nor less than applied comparative pathology, so that if meat-inspection is to be efficient it must be carried out by those who have had some training in that line; this also applies to milk-inspection. Is there any danger from the ingestion of milk from a tuberculous cow? Dr. Peters admits that there is danger from the consumption of milk from a tuberculous cow, and I have no doubt that Dr. Peters has seen a great many that prove to be excessively diseased who seemed to be healthy during life. It is an unquestionable truth that cows that appear to be well can produce bad milk. Cows' milk has been found that killed 13 per cent. of guinea-pigs, showing that the active tubercle bacilli were there. Of course, whether they are numerous enough to infect a person, no one can tell, for no one will use milk of that character.

It is said, by those who are informed on this subject, that there is no case on record where it has been shown conclusively that tuberculosis has been conveyed by the use of milk. That is unquestionably true. There is no case on record where we are able to say positively, but at the same time we know that the germ of tuberculosis in the cow is the same as tuberculosis in man; we know that cows furnish milk containing large numbers of tubercle bacilli, and that in many cases people have tuberculosis in the intestinal tract. When a child from a healthy family contracts tuberculosis and dies, there is often a great deal of circumstantial evidence to uphold our theory, and men have been hung on circumstantial evidence no stronger than what we have.

The experimental work that has been carried on in Massachusetts has gone to show that there is a slight physical difference between the tubercle bacilli of cattle and man. They also show that when these two are injected in cows the bovine is more virulent than the human, and that the germs from the bovine will kill a man quicker than those from human. We do know that where a germ has been grown in a number of generations, it acquires a higher degree of virulence than it has after it has been carried through a few generations. Germs from the bovine source injected into horses will kill quicker than from the human, and we all know that horses are not susceptible to tuberculosis.

We will never know that human beings contract tuberculosis from drinking milk until we are able to take a man and lock him up in a glass cage, using strict antiseptic precautions, and feed him on suspected milk. At the same time we physicians and everybody believe that the dust in rooms that have been occupied by consumptives, and the dust on the street where they have expectorated, are means of spreading the disease, and the Board of Health believe that it is dangerous for consumptives to expectorate on the floors of public conveyances. We have not found a single case where an individual has contracted tuberculosis in that way. So long as we recognize these dangers it is our duty to avoid them, and so long as persons die from tuberculosis, it is the veterinarian's and human physician's duty to remove the causes, which means the prohibition of the sale of meat and milk which is not healthy.

You may think I am assuming too much when I say that you will have meat- and milk-inspection, and you may think so especially because I am a member of Tammany Hall; but I repeat that you will have meat- and milk-inspection in this city carried out by veterinarians, and I say so because we can see this system now growing all over the world. It is a reform that is sure to come, and notwithstanding your experience here in New York, it is stated frequently that reforms never go backward, and this is true of sanitary reforms to a greater extent than it is to political.

The value of meat-inspection has been tested in the most advanced cities of Europe, and it is growing each year more and more. This is not only true of Europe, but it is also true of the United States, for ten years ago there was not a veterinarian employed in this capacity, whereas now there are approximately about one hundred and fifty veterinarians in the Federal Government. There are two in Pennsylvania, another will soon be appointed, and these three will be assisted by two or three laymen, who will no doubt be replaced by veterinarians.

I have no doubt that it will reach New York City, and that when it comes it will find the surroundings congenial, and before long New York will have a thorough meat-inspection carried out by the only men who can do work of this kind—carried out by veterinarians.

Prof. Hickman: I have greatly enjoyed and been very much interested in the paper read by Dr. Gill, the essayist of the evening, and likewise in listening to the remarks of the gentlemen who have followed him.

I have a keen appreciation of the importance of the relation of veterinarians to the public health as inspectors of meat and milk, and while I shall not occupy any more time in speaking of the diseases and conditions which render these animal food-products unfit for human food, I shall direct what I have to say more particularly to the subject, and to the two means by which the qualifications of meat and milk inspectors are acquired. The two means alluded to will be manifest as I proceed.

All things being equal, I think it will be admitted that the knowledge resulting from experience or contact with an object, especially if that object be a living creature, will be of a more practically valuable sort than if attained by promiscuous, general, or special reading. If this proposition is patent, so, likewise, must be the fact that the adaptability and the capabilities of the educated and trained veterinarian render his relations to the public health, as an inspector of meat and milk—the chief articles of food of well-fed humanity—of infinitely closer and greater importance than is possible to members of our sister profession or any other class of individuals; for the veterinarian, by contact and training, is alone fitted as health-mediator and judge of animal products for food-consumption. (Not by any means, however, for the reason advanced by the man who knew all about swine, namely, because he was "raised among them," and yet, I would not for a moment depreciate the advantages of association.)

Having already strayed "from the sublime to the ridiculous," let us allow our thoughts for a moment to dwell upon the æsthetic, and bring within our mental vision, using as an illustration, the golden-rod in the early autumn. Thus, the mental attainment in medical science that is acquired through studious application to books may be compared to the bud; the after-development to the bloom—its fulness, of course, depending upon soil, environment, and the natural vigor of the plant, or, if you please, the talent and persistence of the individual. The golden-rod, as an illustration in point, occurred to my mind because of its being the national flower, and at the same time suggestive in name of the golden rule; for, next in value to the mental attainment of an inspector of animal food-products, would closely follow his practical experience—the result of contact, handling, and association. In fact, I believe it impossible to separate or differentiate the value of these two sources of education and development, since they must be so intimately related that either one without the other would fail to qualify an individual for the performance of the exceedingly important duties of inspector of meat and milk. The family physician gains his most valuable information through hospital and other practice upon subjects of his own race. By these means he learns to apply his knowledge, which knowledge, by association, experience and contact, becomes enhanced; but the very means of his development and the nature of his professional duties debar him, disqualify him, for lines of practical work which must of necessity fit the veterinary physician and surgeon for the functions of his office and the fulfilment of the obligations he has assumed in the interests of science and humanity.

Therefore, let the family physician, the agriculturist, the slaughterer and packer, the milk-producer and vender, attend to his respective duties as such, leaving the determination of the fitness of animals and their products for food consumption to the veterinarian, who devotes his life, his abilities, and his energies to the acquirement of such wisdom and the execution of such obligations as devolve upon him, which, because of evident circumstances and conditions, make him a true savant in questions of hygiene as pertaining to domesticated animals, and their products, and their relative values, uses and fitness for human food.

It is true that medical science (I use the term advisedly, for the greater number of its varied branches are true sciences) has made its advancement chiefly through experimentation upon the lower animals. It is probably equally true that both branches are advancing in equal ratio. The results of such experimentation, however, as applied to the veterinary branch of medicine, lead to knowledge that is more or less positive, while, as applied to the other branch, and the *genus homo*, it can be but relative.

I think it has been clearly shown that the work of meat- and milk-inspection belongs exclusively to the veterinarian, and likewise the importance of having qualified representative members of the profession in every State or municipal Board of Health. I am sorry that this claim is not more generally recognized, and that it is possible and pertinent for such editorial squibs to appear as I noticed in the February 5th *Rural New Yorker*—*i. e.*, "The New York State Board of Health has been expending a little money in testing the dairy herds at State institutions. This serves to protect the inmates from diseased milk, and sets a good example. . . . While we are thinking about the State Board of Health, it occurs to us that a veterinarian or two would not be out of place upon it. Very likely it may have to pass upon the fitness of men for herd-testing, and physicians do not get such training in comparative medicine as would be useful in that event."

I believe it just, proper, and essential that the veterinary profession have representation in every scientific body, State, municipal, corporate, or otherwise, where the subject of the *relation* of the diseases of man and animals, or the fitness of animal products for human food is investigated and passed upon; and I believe that such bodies or associations of men will make more rapid advancement by such accessions to their organizations; and, finally, I am of the opinion that none but veterinarians should act in the capacity of inspectors of animals and their products, such as meat and milk.

Dr. Lowe: I have been very much interested in Dr. Gill's paper and so far as the other addresses went.

This subject of meat-and milk-inspection is certainly a very important one, and veterinarians have to go into it carefully, slowly, and, of course, thoroughly. People will have to be educated up to what veterinary science will do for them.

In speaking of meat-and milk-inspection most of the speakers dwelt on tuberculosis; but I think our laws in regard to the inspection of diseases of animals should be general, and applied to all the diseases that are communicable from the animal to mankind, and not to tuberculosis alone; but no doubt tuberculosis is the most important that we have at the present time. I think we all recollect when pleuro-pneumonia was so prevalent, and all investigations were directed in that direction.

Any of us that may have any influence in shaping what is to be done in that line know that we must have legislation, not directed, toward tuberculosis but all animal diseases.

I think no great results will be reached by destroying infected animals until we pay more attention to breeding and to sanitary conditions. Carelessness in this line often produces the same disease or some other diseases, and we all know that the sounder the animal the better able it is to resist infection. It is well known, I think, that every man who drinks tuberculous milk and eats tuberculous meat does not contract tuberculosis. There is a great deal to accomplish in the line of breeding which largely depends on more careful selection of the stock, for sometimes one infected bull will cause a great deal of harm.

In New Jersey we have labored under considerable disadvantage by reason of not having legislation. The State Board of Health has control over all diseases of animals; then we have the Dairy Commission, and we have a Tuberculosis Commission vested with full power to deal with tuberculosis; therefore, if a man has a diseased animal he has to spend a good deal of time in finding out which one he has to consult.

A practical and experienced veterinarian should examine animals first, then the power of the other commissions could be applied. Not very long ago we had a typhoid-fever epidemic in Paterson, N. J., and there were a great many deaths. The investigation traced it to a certain dairy in Sussex County, where it was found that they had what was called a case of walking typhoid fever. This milk was shipped to different parts of the State, and by lack of facilities could not be traced.

Dr. W. Horace Hoskins: The subject which is open for consideration, and which has been so well presented by Dr. Gill, is one of great importance. This subject is one that is pressing its importance upon the people of this country, and it is well that we who are so directly interested in this work should be foremost in consideration of all the measures that are destined to lead us out of the present unsatisfactory conditions, and especially the very important ones of meat and milk, in which we are very much interested, and which by our education and training are so well fitted to solve the questions relative thereto in a way that shall be satisfactory in the largest measure to all concerned.

The question of meat- and milk-inspection is probably the most important one that presents itself for consideration to the United States and the whole world, and it has fallen to you to bring this matter to the more direct attention of the authorities of your State and especially of your city. In this, because of your ability and of your training, and as your duty as citizens as well as professional men, you should be interested. I was surprised in looking over some of the recent statistics to find that New York State, with her great animal-industry interests, that the subject of milk- and meat-question, which constitutes one of the leading industries, that the authorities have not been able to find profitable employment of more money to help solve some of these questions, and do her part of the work that is going to establish all over our country a thorough food-inspection. We should not confine it to meat which goes abroad, for our own interest is greater than any foreign, and of far more importance than the better physical development of nations three thousand miles away. Your own statistics show that in seventeen years there has been less than twenty thousand dollars spent in the employment of veterinary services for the inspection of diseases incidental to your live-stock interests in the State of New York; surely a paltry sum. She must look to the veterinary profession to lead in this work, and if New York State has found it necessary to spend so small an amount of money, it is that she has not realized how great is her responsibility in guarding human life. So far as this subject relates to our profession, it is our duty to educate public opinion and arouse public sentiment. The avenues of education are multiple in number. However, every citizen appreciates more or less the dangers there are in the supply of milk, not alone from the animals, but from conditions surrounding its production and that are incidental to its proper delivery to the consumer.

It is equally well known how susceptible milk is to the presence of other products, and, therefore, of the special care that must be taken to prevent it from being contaminated.

We as veterinarians know particularly of the very many dangers there are: We know the different kinds of milk; we know of the results of bad sanitary conditions of stables; we know the care needed for animals; we know all the dangers of food, and how important they are for a good milk-supply. We cannot hope to obtain an ideal system at first, but that is something which is sure to come in time. I think it is a sad state of affairs if a city cannot regulate its own food-products and control the introduction of only good and wholesome foods for her people.

It is certain that the establsihment of a system of meat- and milk-inspection is needed, and that we must lead in trying to formulate regulations for this work. In Massachusetts and Pennsylvania this work has been going on, not as rapidly as we would like, but the education of the people is being advanced daily.

It is the purpose of this meeting to bring this very important subject more forcefully to the attention of the people of your city and State, and I am sure that much good will flow from this movement.

